# Homochiral and racemic MicroED structures of a peptide repeat from the ice-nucleation protein InaZ

**DOI:** 10.1107/S2052252518017621

**Published:** 2019-01-24

**Authors:** Chih-Te Zee, Calina Glynn, Marcus Gallagher-Jones, Jennifer Miao, Carlos G. Santiago, Duilio Cascio, Tamir Gonen, Michael R. Sawaya, Jose A. Rodriguez

**Affiliations:** a Department of Chemistry and Biochemistry, UCLA-DOE Institute for Genomics and Proteomics, University of California Los Angeles, Los Angeles, CA 90095, USA; b Department of Biological Chemistry, UCLA-DOE Institute for Genomics and Proteomics, University of California Los Angeles, Los Angeles, CA 90095, USA; c Howard Hughes Medical Institute, Departments of Physiology and Biological Chemistry, University of California Los Angeles, Los Angeles, CA 90095, USA

**Keywords:** amyloid, racemic, electron diffraction, ice nucleation, intermolecular interactions, co-crystals, electron crystallography, structural biology

## Abstract

The ice-nucleation protein InaZ from *Pseudomonas syringae* contains a large number of degenerate repeats that span more than a quarter of its sequence and include the segment GSTSTA. *Ab initio* structures of this repeat segment, resolved to 1.1 Å by microfocus X-ray crystallography and to 0.9 Å by the cryo-EM method MicroED, were determined from both racemic and homochiral crystals. The benefits of racemic protein crystals for structure determination by MicroED were evaluated and it was confirmed that the phase restriction introduced by crystal centrosymmetry increases the number of successful trials during the *ab initio* phasing of the electron diffraction data. Both homochiral and racemic GSTSTA form amyloid-like protofibrils with labile, corrugated antiparallel β-sheets that mate face to back. The racemic GSTSTA protofibril represents a new class of amyloid assembly in which all-left-handed sheets mate with their all-right-handed counterparts. This determination of racemic amyloid assemblies by MicroED reveals complex amyloid architectures and illustrates the racemic advantage in macromolecular crystallography, now with submicrometre-sized crystals.

## Abstract 2

The atomic asymmetry, left- or right-handedness, that is present in macromolecules and that was first described by Pasteur in his experiments with tartaric acid is evident even in complex molecular assemblies such as amyloid fibrils. Here, using the cryo-EM method MicroED, it is shown that a segment from the ice-nucleation protein InaZ assembles into homochiral and racemic water-binding amyloid protofibrils.

## 1. Introduction   

Expressed by a subset of microorganisms, ice-nucleation proteins are capable of stimulating ice formation in supercooled water (Green & Warren, 1985 ▸). The Gram-negative microbe *Pseudomonas syringae* is sold commercially as Snomax^®^ for its ice-nucleating activity (Green & Warren, 1985 ▸; Cochet & Widehem, 2000 ▸). The ice-nucleation protein InaZ is produced by *P. syringae* and localized to its outer membrane (Green & Warren, 1985 ▸; Wolber *et al.*, 1986 ▸). The sequence of InaZ is approximately 1200 residues in length, over half of which includes degenerate octapeptide repeats. A sub­population of degenerate repeats share the consensus motif GST*X*T(A/S), where *X* represents an unconserved amino acid (Supplementary Fig. S1; Green & Warren, 1985 ▸; Warren *et al.*, 1986 ▸). These repeats are shared by other Ina proteins and may collectively contribute to ice nucleation (Green & Warren, 1985 ▸; Kobashigawa *et al.*, 2005 ▸; Han *et al.*, 2017 ▸).

Despite the crystallographic determination of structures of other ice-binding proteins (Davies, 2014 ▸; Garnham, Campbell & Davies, 2011 ▸), InaZ remains recalcitrant to crystallization. Models of full-length InaZ have proposed it to have a β-helical (Garnham, Campbell, Walker *et al.*, 2011 ▸; Graether & Jia, 2001 ▸) or solenoid-like fold rich in stacked β-strands (Cochet & Widehem, 2000 ▸; Pandey *et al.*, 2016 ▸). These features are shared by amyloid filaments: their tightly mated β-sheets form fibrils that can cross-link, cluster and be functional (Nelson *et al.*, 2005 ▸; Sawaya *et al.*, 2007 ▸; Fitzpatrick *et al.*, 2017 ▸; Eisenberg & Jucker, 2012 ▸; Maury, 2009 ▸). Functional amyloid assemblies appear across the tree of life (Wasmer *et al.*, 2008 ▸; Hughes *et al.*, 2018 ▸; Maury, 2009 ▸; Tayeb-Fligelman *et al.*, 2017 ▸) and can contain low-complexity regions with degenerate repeats (Hughes *et al.*, 2018 ▸).

Success in determining amyloid structures was first achieved by crystallizing short segments that stabilize the cores of fibrils through a motif known as the steric zipper (Nelson *et al.*, 2005 ▸; Sawaya *et al.*, 2007 ▸). However, the propensity of elongated β-strands to twist or kink can limit crystal growth, sometimes yielding nanocrystals that pose a challenge for structure determination (Rodriguez *et al.*, 2015 ▸). These limits have recently been overcome in part by the development of the cryo-electron microscopy (cryo-EM) method, electron microdiffraction (MicroED; Shi *et al.*, 2013 ▸). MicroED yields high-resolution structures from protein crystals no thicker than a few hundred nanometres (Shi *et al.*, 2016 ▸; Rodriguez *et al.*, 2017 ▸). Because of this, MicroED has helped in determining the structures of a number of amyloid protofibrils (Rodriguez *et al.*, 2015 ▸; Krotee *et al.*, 2017 ▸) at atomic resolution, some *ab initio* (Sawaya *et al.*, 2016 ▸; Gallagher-Jones *et al.*, 2018 ▸).

Racemic crystallography further facilitates the crystallization of proteins and peptides (Matthews, 2009 ▸; Yeates & Kent, 2012 ▸; Patterson *et al.*, 1999 ▸), including ice-binding proteins (Pentelute *et al.*, 2008 ▸). Mixing left-handed (l) and right-handed (d) enantiomers of a macromolecule improves its likelihood of crystallization and facilitates structural analysis (Yeates & Kent, 2012 ▸; Wukovitz & Yeates, 1995 ▸). Crystallographic phases are restricted for data from centrosymmetric crystals, making the phase problem associated with the determination of their structure more tractable (Yeates & Kent, 2012 ▸). This is advantageous for structure determination by direct methods (Hauptman, 1986 ▸), where phases must be computed from measured intensities alone (Hauptman, 1986 ▸, 2001 ▸; Sheldrick, 2008 ▸). Accordingly, various polypeptide structures have been determined by racemic X-ray crystallo­graphy, including those of ester insulin, plectasin and an antifreeze protein (Pentelute *et al.*, 2008 ▸; Avital-Shmilovici *et al.*, 2013 ▸; Mandal *et al.*, 2009 ▸, 2012 ▸). While the benefits of racemic crystallography are evident for X-ray diffraction (Matthews, 2009 ▸), questions remain about the potential for exploiting these benefits in MicroED.

Hypothesizing that the repeat segments of the ice-nucleation protein InaZ may form amyloid-like assemblies, we set out to interrogate the structure of GSTSTA from both homochiral and racemic crystals by MicroED. In doing so, we also assessed the fidelity of MicroED data in racemic structure determination. By comparing the structures of homochiral and racemic GSTSTA, we gauge the effect of racemic self-assembly on protofibril architecture. With these structures of a core repeat in the InaZ protein, we begin an atomic-level investigation of peptide segments derived from ice-nucleation proteins (Pandey *et al.*, 2016 ▸).

## 2. Methods   

### 2.1. Sequence analysis of ice-nucleation proteins   

The sequence of the ice-nucleation protein InaZ from *P. syringae* was screened for the existence of hexameric degenerate repeat motifs that contained one or more threonine residues (Supplementary Fig. S1). The repeats were then evaluated for their propensity for amyloid fibril formation by ZipperDB (Supplementary Fig. S1). For each, a Rosetta energy score was calculated. A single repeat, GSTSTA, was chosen from this list of hexameric sequences. This segment appears five times identically in the sequence of InaZ, first at residue 707, and is part of a group with the consensus motif GST*X*T(A/S) that appears 59 times in the InaZ sequence.

### 2.2. Synthesis, purification, characterization and crystallization of l- and d-enantiomers of the InaZ-derived peptide GSTSTA   

The l-enantiomer of GSTSTA was purchased from GenScript with 98% purity. The d-enantiomer of GSTSTA was synthesized by solid-phase peptide synthesis and was purified using a Waters Breeze 2 HPLC System in reversed phase buffered with trifluoroacetic acid (Supplementary Fig. S2). The two enantiomers were qualified by ESI-MS on a Waters LCT Premier. The spectrum of the l-enantiomer showed an [*M*+H]^+^ peak of 523.30 g mol^−1^ (expected 523.22 g mol^−1^) and a dimer [*M*+*M*+H]^+^ peak of 1045.6 g mol^−1^ (expected 1045.44 g mol^−1^). The spectrum of the d-enantiomer showed an [*M*+H]^+^ peak of 523.24 g mol^−1^ (expected 523.22 g mol^−1^) and a dimer [*M*+*M*+H]^+^ peak of 1045.49 g mol^−1^ (expected 1045.44 g mol^−1^) (Supplementary Fig. S2).

Crystals of l-GSTSTA were grown as follows. Lyophilized peptide was weighed and dissolved in ultrapure water at concentrations of between 82 and 287 m*M*, assuming a 1:1 ratio of peptide to trifluoroacetic acid (TFA) in the lyophilized powder. Crystals were grown at room temperature by the hanging-drop method in a high-content 96-well Wizard screen. Of the many crystallization trials that yielded crystals, those obtained from a condition consisting of 0.1 *M* CHES buffer pH 9.5, 10%(*w*/*v*) PEG 3000 were used for microfocus X-ray data collection. Another promising condition was optimized by the hanging-drop method in 24-well plates. This condition consisted of 0.1 *M* McIlvaine (citrate–phosphate) buffer pH 4.2, 12.5%(*w*/*v*) PEG 8000, 0.1 *M* sodium chloride and was used to grow crystals of l-GSTSTA in batch.

Crystals of racemic GSTSTA were grown as follows. Lyophilized powders of l-GSTSTA and d-GSTSTA were separately weighed and dissolved in ultrapure water so that the concentrations of the two enantiomers matched. Crystal formation was screened at concentrations ranging from 82 to 123 m*M* after accounting for TFA. Control trays containing only l- or d-GSTSTA were prepared simultaneously alongside racemic screens. All three trays were stored and monitored at room temperature, with crystal formation observed in various conditions. Images of every well were collected after 3 h, one day, three days, five days and seven days, and crystal formation was monitored over time. A condition consisting of 0.1 *M* imidazole pH 8.0, 10%(*w*/*v*) PEG 8000 produced the best crystals.

Crystals were batch grown for data collection by MicroED. Lyophilized l-GSTSTA peptide was weighed and dissolved in 0.1 *M* McIlvaine (citrate–phosphate) buffer pH 4.2, 12.5%(*w*/*v*) PEG 8000, 0.1 *M* sodium chloride to an effective final concentration of 123 m*M*, mimicking the final concentration of a hanging drop in the 24-well optimization. Lastly, the solution was seeded with crystal needles extracted from crystals grown in the 24-well optimization described above. Batch crystals of racemic GSTSTA were grown from lyophilized l-GSTSTA and d-GSTSTA that had been separately weighed and dissolved in 0.1 *M* imidazole buffer pH 8.0 containing 10%(*w*/*v*) PEG 8000 to a final concentration of 50 m*M* for each enantiomer after accounting for the mass contributed by TFA.

### 2.3. Microfocus X-ray data collection and structure determination   

Crystals of l-GSTSTA were harvested from a 96-well hanging drop using MiTeGen loops and flash-cooled in liquid nitro­gen. No additional cryoprotectant was used other than the PEG 3000 that was already present in the mother liquor. 72 diffraction images were collected with an oscillation range of 5° from a single crystal; 40 of these were indexed and integrated. Crystals of racemic GSTSTA were harvested from a 96-well hanging drop using MiTeGen loops and flash-cooled in liquid nitrogen. No additional cryoprotectant was used other than the PEG 8000 that was already present in the buffer. 144 diffraction images were collected with an oscillation range of 2.5° from a single crystal; 64 of these were indexed and integrated.

Diffraction data were collected from both homochiral and racemic GSTSTA crystals under cryogenic conditions (100 K) on beamline 24-ID-E at the Advanced Photon Source (APS) equipped with an ADSC Q315 CCD detector using a 5 µm beam with wavelength 0.979 Å. Signal was only limited by the edge of our detector at approximately 1.1 Å; as such, higher resolution data could perhaps be achieved by modifying the experimental geometry and/or adjusting the energy of the X-ray beam in the experiment. Data from both homochiral and racemic crystals were reduced in *XDS* (Kabsch, 2010 ▸) and yielded *ab initio* solutions using *SHELXT* and *SHELXD* (Sheldrick, 2015 ▸). The phases obtained from these coordinates produced maps of sufficient quality for subsequent model building in *Coot* (Emsley *et al.*, 2010 ▸). The resulting models were refined against the measured data using *PHENIX* (Adams *et al.*, 2010 ▸).

### 2.4. Electron microscopy, MicroED data collection and structure determination   

Crystals were prepared for MicroED data collection following a variation of the procedures detailed in Rodriguez *et al.* (2015 ▸) as follows. Following a 1:2 dilution in ultrapure water, crystals were applied onto glow-discharged grids of type (PELCO easiGlow) 300 mesh Cu 1/4. Excess liquid was blotted off onto filter paper wetted with 4 µl ultrapure water to avoid salt-crystal formation. Grids were plunge-frozen into liquid ethane using a Vitrobot (FEI). Grids were then initially stored in liquid nitrogen before being transferred to a liquid-nitrogen-cooled Gatan 626 cryo-holder for insertion and manipulation within the electron microscope.

MicroED data were collected from three submicrometre-thick needle crystals of l-GSTSTA and two submicrometre-thick needle crystals of racemic GSTSTA. Briefly, frozen hydrated crystals of either l-GSTSTA or racemic GSTSTA were visually inspected in overfocused diffraction mode on a cryocooled FEI Tecnai F20 microscope operated at 200 kV (Janelia Research Campus). The diffraction patterns used for structure determination were collected on a TVIPS TemCam-F416 CMOS detector in rolling-shutter mode. For l-GSTSTA, diffraction patterns were collected during unidirectional rotation with 2 s exposures. For racemic GSTSTA, diffraction patterns were collected during unidirectional rotation with 3 s exposures. A rotation rate of 0.30° s^−1^ and rotation angles ranging from −63° to 72° were used for both. Beam intensity was held constant, with an average dose rate of 0.003–0.005 e^−^ Å^−1^ s^−1^ or ∼0.01 e^−^ Å^−2^ per image, corresponding to a total dose of ∼1–3 e^−^ Å^−2^ per data set. Data were recorded at an effective camera length of 730 mm, which is the equivalent of a sample-to-detector distance of 1156 mm in a corresponding lenseless system. All diffraction was performed using a circular selected area aperture of ∼1 µm^2^ in projection.

Diffraction movies were converted to the SMV file format using TVIPS tools as described previously (Hattne *et al.*, 2015 ▸). Indexing and integration were performed in *XDS*. Partial data sets from three l-GSTSTA crystals were sorted and merged in *XSCALE*. Intensities from a total of 196 diffraction images were merged. An *ab initio* solution was achieved using *SHELXD* (Sheldrick, 2015 ▸). To achieve a complete data set from racemic GSTSTA crystals, the integrated diffraction intensities from partial data sets of two different crystals were sorted and merged in *XSCALE*. Intensities from a total of 145 diffraction images were merged. An *ab initio* solution was achieved using *SHELXD* and *SHELXT* (Sheldrick, 2015 ▸). Although *XDS* accurately differentiated the Laue classification for the racemic GSTSTA data, *SHELXT*, which does not rely on user input for space-group selection, ensured a correct solution for the racemic data. *SHELXT* selected *P*2_1_/*c* as the racemic space group, a choice corroborated by the systematic absences that were present in the data. The phases obtained from the l-GSTSTA and racemic GSTSTA coordinates produced by *SHELX* were used to generate maps of sufficient quality for subsequent model building in *Coot* (Emsley *et al.*, 2010 ▸). The resulting models were refined with *PHENIX* (Adams *et al.*, 2010 ▸), using electron scattering form factors, against the measured data.

### 2.5. Analysis of homochiral and racemic GSTSTA structures   

In the analysis of the hydrogen-bonding and assembly inter­actions of each l-GSTSTA structure, an assembly of four strands, composed of two pairs in mating sheets, was used to find all unique hydrogen bonds, while racemic GSTSTA required an assembly of 12 strands composed of three strands from a pair of mating sheets and six more strands related by translation along the protofibril axis to achieve a unique set of hydrogen bonds. Hydrogen bonds were tabulated from this structure using *HBPLUS* (McDonald & Thornton, 1994 ▸).

Distances between strands along a sheet were calculated as differences between α carbons of one strand and its neighbor along the same sheet. These distances were calculated for both GSTSTA and GNNQQNY using PDB entry 1yjp (Sawaya *et al.*, 2016 ▸). The angle between a strand and its corresponding sheet was calculated against the plane formed by α carbons along that sheet.

### 2.6. Analysis of phases in structures determined by MicroED and X-ray crystallography   

To analyze the distribution of phases associated with reflections measured from racemic crystals by both X-ray and electron diffraction, data reduction was performed in space group 1 (*P*1) and refined in *PHENIX* against a model encompassing the entire unit cell of four strands. This model was obtained by applying all symmetry operations on the asymmetric unit of the *P*2_1_/*c* structure. Refinement in *P*1 allowed symmetry to be broken, no longer restricting phases to 0 or 180°, as the phases changed in the case where coordinates deviated from their symmetry-related positions. The resulting set of reflections and phases were analyzed in *MATLAB*. We plotted the observed and calculated magnitudes of each reflection against each other and the set fitted by linear regression. For each measured magnitude, the associated phases were plotted and showed a bimodal distribution. Histograms were drawn using these data to evaluate phase distributions; the standard deviation of these was computed by merging the distributions around 0 and 180° using a modulo operation.

### 2.7. Analysis of paired reflections in MicroED and X-ray crystallographic data   

Merged data sets collected by either MicroED or microfocal X-ray crystallography were paired for homochiral and racemic crystals of GSTSTA. MicroED data .mtz files were scaled against their corresponding X-ray counterparts, where corresponding reflections were paired and missing reflections were ignored within a single .mtz file. This was achieved using custom scripts and the *RSTATS* program, which scaled and compared common reflections between corresponding data sets. The corresponding distributions of Fourier magnitudes were then analyzed using *MATLAB*, in which a best-fit line was determined for each of the paired data sets. Zones were visualized using the *HKLVIEW* program, in which either *h*, *k* or *l* were selectively set to zero.

## 3. Results   

### 3.1. Identification, synthesis and crystallization of amyloid-forming ice-nucleation protein (INP) segments   

With the goal of characterizing the structural properties of degenerate repeats in INPs, we identified a group of hexapeptides within the set of InaZ repeats and evaluated their amyloid-forming propensities (Goldschmidt *et al.*, 2010 ▸; Supplementary Fig. S1). We ranked the hexapeptides based on their predicted propensity for amyloid zipper formation, their repeated appearance in INP sequences and whether they contained polar residues, including threonine (Supplementary Fig. S1). We chose to further investigate a segment whose sequence, GSTSTA, appears identically five times within InaZ at residues 707–712, 755–760, 803–808, 851–856 and 899–904. For simplicity, we numbered the segment 707–712.

We evaluated the crystallization potentials of synthesized l- and d-enantiomers of GSTSTA (Supplementary Fig. S2) compared with that of their racemic mixture by performing high-throughput crystallization trials and monitoring crystal growth. Most crystals appeared within two weeks of the start of each trial. In some conditions crystallization was observed as early as 3 h after the start of the trial. Racemic mixtures produced a greater number of successful crystallization conditions across a broad variety of trials (Supplementary Fig. S3). The number of successful conditions that were identified for racemic mixtures outpaced those identified for each enantiomer alone (Supplementary Fig. S3), which is consistent with previous predictions (Yeates & Kent, 2012 ▸). In conditions in which both racemic and single-enantiomer crystals grew, racemic crystals appeared sooner (Supplementary Fig. S3). Minor differences in the speed of crystal appearance and the total number of conditions with identifiable crystals were also seen between l- and d-enantiomers. Fewer conditions were found to produce d-enantiomer crystals across all trials (Supplementary Fig. S3). These differences may have been a consequence of subtle inequities in the amount of residual trifluor­acetic acid (TFA) associated with each enantiomer in lyophilized powders. These effects may have been magnified by the relatively high concentrations of peptide required for crystallization of these segments (∼100–150 m*M*).

The crystallization conditions chosen for structure determination of homochiral GSTSTA (l-GSTSTA) and racemic GSTSTA (dl-GSTSTA) yielded a high density of well ordered microcrystals, each with a unique powder diffraction pattern, indicating they had formed distinct structures (Supplementary Fig. S4). Microcrystals were optimized from these conditions for microfocal X-ray diffraction; unoptimized batch conditions yielded nanocrystal slurries that were directly suitable for MicroED. Since the powder diffraction patterns of homochiral GSTSTA crystals were identical for both enantiomers (Supplementary Fig. S4), we focused our investigation on the l-enantiomer.

### 3.2. *Ab initio* structure determination of l-GSTSTA   

**Figure 1:**
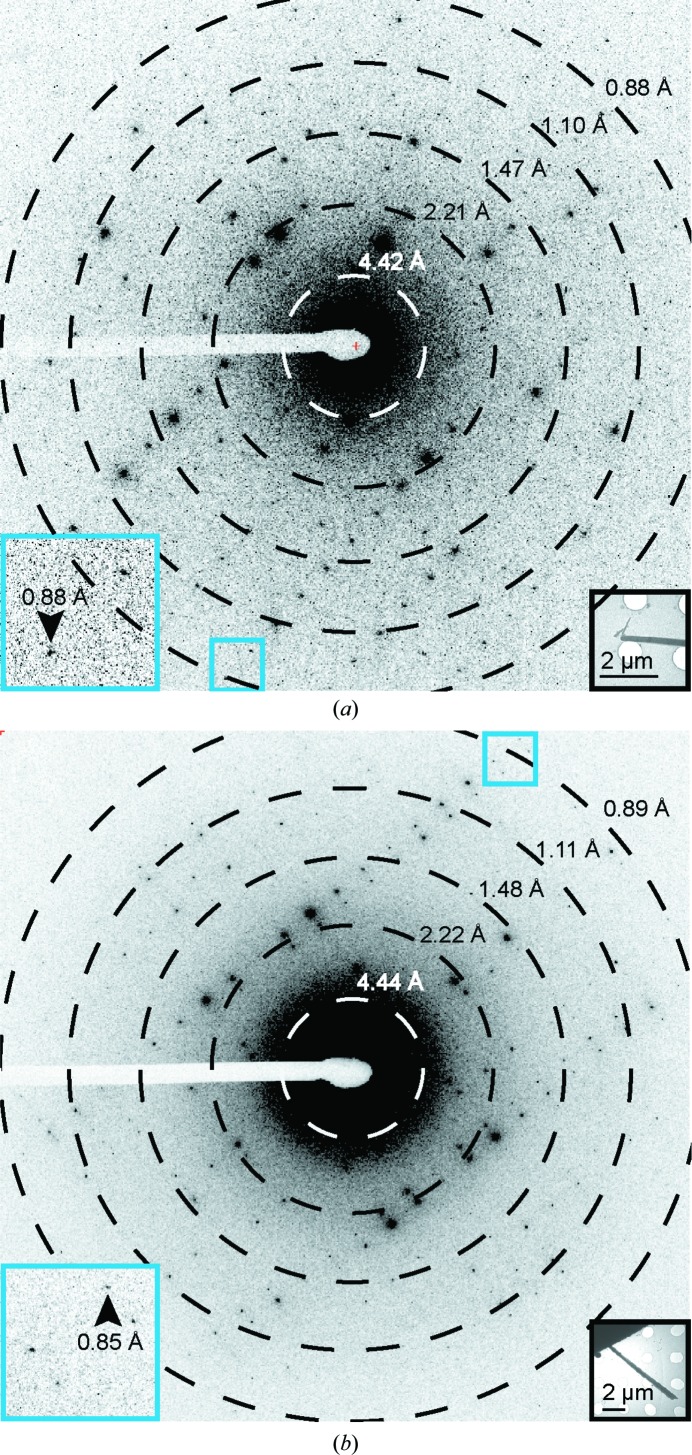
Single diffraction patterns of homochiral l-GSTSTA (*a*) and racemic GSTSTA (*b*) measured during continuous-rotation MicroED data collection. Each pattern corresponds to a 0.6° wedge (*a*) or a 0.9° wedge (*b*) of reciprocal space. Black insets show overfocused diffraction images of the crystals used for diffraction; blue squares correspond to magnified regions (blue insets) of the pattern that show diffraction at sub-0.9 Å resolution (black arrows). Resolution circles are indicated by rings; scale bars are 2 µm in length.

We optimized crystals of l-GSTSTA for microfocal X-ray diffraction, starting from dense needle clusters and ending with single needles (Supplementary Fig. S5). Crystals grown in batch were monodisperse rods of 1–10 µm in length and 100–500 µm in width; these diffracted to approximately 0.9 Å resolution by MicroED (Fig. 1 ▸). X-ray diffraction from a single crystal of l-GSTSTA yielded a 91.7% complete data set to approximately 1.1 Å resolution (Supplementary Table S1), while data sets from three crystals of l-GSTSTA obtained by MicroED were merged to achieve a data set with an overall completeness of 86.4% at 0.9 Å resolution. It is important to note that the X-ray data in this case were limited by the detector geometry, which could be adjusted to facilitate slightly higher resolution. Atomic structure solutions were determined for l-GSTSTA from both microfocal X-ray and MicroED data by direct methods (Sheldrick, 2008 ▸; Supplementary Fig. S6).

**Figure 2:**
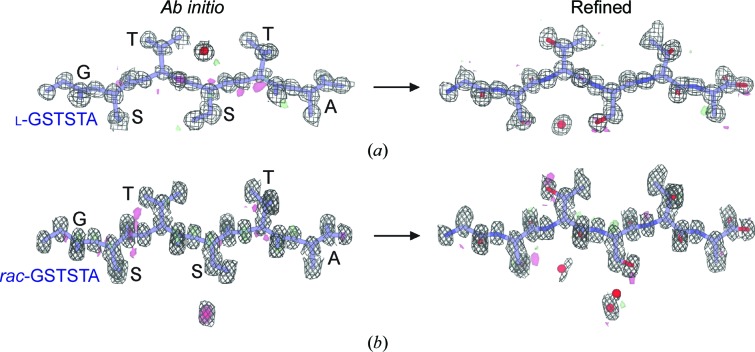
*Ab initio* structures and electrostatic potential maps of l-GSTSTA (*a*) and racemic GSTSTA (*b*). Each map in (*a*) is overlaid onto the initial atomic coordinates calculated by *SHELXD* from MicroED data. Each map in (*b*) is overlaid onto its corresponding refined model. The 2*F*_o_ − *F*_c_ map represented by the black mesh is contoured at 1.2σ. Green and red surfaces represent *F*_o_ − *F*_c_ maps contoured at 3.0σ and −3.0, respectively. Modeled waters are present as red spheres. The waters modeled in the *ab initio* solution in (*a*) and the refined structure in (*b*) are related by symmetry.

After 50 000 trials, *SHELXD* yielded correlation figures of merit (CFOMs) of greater than 80 for both X-ray diffraction and MicroED data (Supplementary Fig. S6; Sawaya *et al.*, 2016 ▸). The initial l-GSTSTA solution with the highest CFOM shows 33 atoms for the X-ray data set and 36 atoms for the MicroED data set (Fig. 2 ▸ *a* and Supplementary Fig. S7). During refinement, the number of atoms in the X-ray structure increased to 36 peptide atoms and one bound water (Supplementary Fig. S7). The final solution achieved from the 0.9 Å resolution MicroED data also contained 36 atoms in the peptide chain and one water molecule (Fig. 2 ▸ *a*).

### 3.3. *Ab initio* structure determination of racemic GSTSTA from centrosymmetric crystals   

Like the enantiomerically pure crystals of GSTSTA, crystals of racemic GSTSTA started as dense needle clusters and were optimized to single needles, and diffracted as single crystals on a microfocal X-ray source (Supplementary Fig. S5). Batch crystals of racemic GSTSTA were also rod-shaped and were several micrometres in length and a few hundred nanometres in thickness (Fig. 1 ▸). These were immediately suitable for MicroED and diffracted to approximately 0.9 Å resolution (Fig. 1 ▸). Data from a single crystal obtained by X-ray diffraction produced a 93.7% complete data set at 1.1 Å resolution, while MicroED data from two nanocrystals of racemic GSTSTA were merged to reach an overall complete­ness of 77.4% at 0.9 Å resolution (Supplementary Table S1). Initial atomic structure solutions for racemic GSTSTA were obtained by direct methods (Sheldrick, 2008 ▸; Fig. 2 ▸ *b* and Supplementary Fig. S7).

As with l-GSTSTA, solutions for the racemic crystals yielded correlation figures of merit (CFOMs) of greater than 80 after 50 000 trials (Supplementary Fig. S6). A comparison of the racemic GSTSTA and l-GSTSTA data sets indicated that a higher number of potentially correct solutions were found for the racemic GSTSTA data (Supplementary Fig. S6). The MicroED data show a distribution of CFOM values that is shifted towards higher values, even when truncated to 1.1 Å resolution to match the resolution of the X-ray data sets. However, the most dramatic shift in this distribution is evident at 0.9 Å resolution (Supplementary Fig. S6).

**Figure 3:**
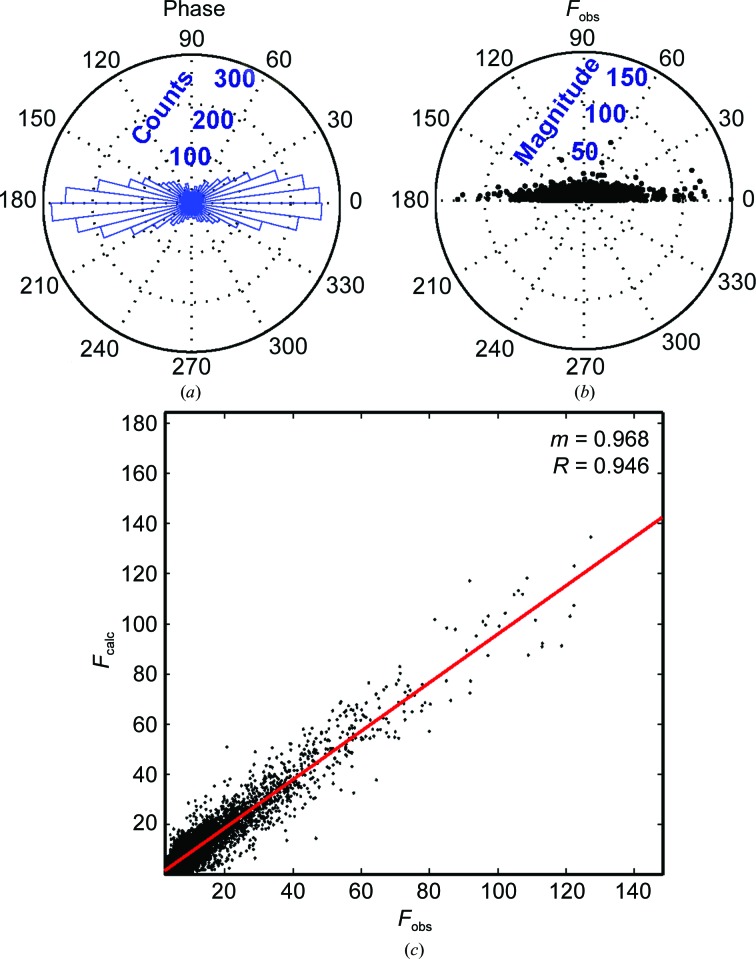
(*a*) The calculated phase associated with each reflection in the *P*1 refinement of racemic GSTSTA data obtained by MicroED was analyzed and plotted as a histogram along the unit circle. (*b*) The magnitude of each reflection is plotted as a function of the absolute value of its associated phase. (*c*) A plot of *F*_o_ versus *F*_c_ values for each reflection in this data set shows a distribution that can be fitted by linear regression, shown as a red line, with slope *m* = 0.97 and *R* value 0.95.

Initial solutions with the highest CFOM show a total of 35 peptide atoms and four waters for the structure determined from X-ray data, and a total of 36 peptide atoms and one water for that determined by MicroED (Fig. 2 ▸ *b*). During refinement, the number of peptide atoms in the X-ray structure increased to 36 (Supplementary Fig. S7), while the MicroED structure gained two waters (Fig. 2 ▸ *b*). Linear regression of observed to calculated structure factors for the MicroED data shows an *R* value of 0.94 and a slope of 0.97 for data reduced in space group *P*1 (Fig. 3 ▸ *c*). These values are in good agreement with those obtained by microfocal X-ray diffraction (Supplementary Fig. S11*c*) and indicate a good fit between model and measurement for the racemic GSTSTA structure.

### 3.4. Paired comparison of Fourier magnitudes measured by X-ray crystallography or MicroED   

A comparison between the X-ray and MicroED data sets for homochiral crystals of GSTSTA shows that these two types of measurement are in close agreement (Supplementary Figs. S8 and S9), although slightly higher merge errors are observed in the MicroED data across resolution bins (Supplementary Table S2). A direct comparison of Fourier magnitudes for paired reflections between these data sets is fitted by a line with a slope of 0.921 and an *R* value of 0.826 (Supplementary Fig. S8). In contrast, the comparison between X-ray and MicroED data for racemic GSTSTA shows a greater difference between the two sets and a lower *R* value for the best-fit line comparing the Fourier magnitudes of paired X-ray and MicroED reflections (Supplementary Figs. S8 and S10). This difference is likely to be owing to a lack of isomorphism between the unit cells of the racemic GSTSTA crystals used for MicroED data collection versus X-ray data collection. The unit-cell parameters for racemic GSTSTA crystals obtained by MicroED and microfocal X-ray crystallography were *a* = 15.23, *b* = 9.29, *c* = 21.06 Å, α = 90.0, β = 108.2, γ = 90.0° and *a* = 14.03, *b* = 9.22, *c* = 20.77 Å, α = 90.0, β = 104.5, γ = 90.0°, respectively (Supplementary Table S1).

### 3.5. Phase restriction in centrosymmetric crystals evaluated by MicroED   

Data from racemic GSTSTA crystals obtained by MicroED and reduced in the centrosymmetric space group *P*21/*c* satisfy refinement with imposed phases of 0 or 180°. The refinement of data from the same crystals reduced in space group *P*1 results in similar residuals to those obtained for space group *P*21/*c* (Supplementary Table S1). The phases that result from refinement of this structure against data reduced in space group *P*1 appear to be bilaterally distributed around 0 and 180° (Fig. 3 ▸ *a*). Collapse of this bimodal phase distribution around *n*π yields a standard deviation of 34.3° (Fig. 3 ▸). When the same procedure is applied to data collected from racemic GSTSTA crystals by X-ray diffraction, a similar trend appears: a normal distribution around *n*π with a standard deviation of 34.4° (Supplementary Fig. S11). Bragg reflections that appear in disallowed regions of phase space (90 and 270°) for both MicroED and X-ray diffraction data are generally weakest (Fig. 3 ▸ and Supplementary Fig. S11). This suggests that the primary source of phase error in MicroED data, as with X-ray diffraction, may come from noisy or weak reflections.

### 3.6. Structure of l-GSTSTA   

**Figure 4:**
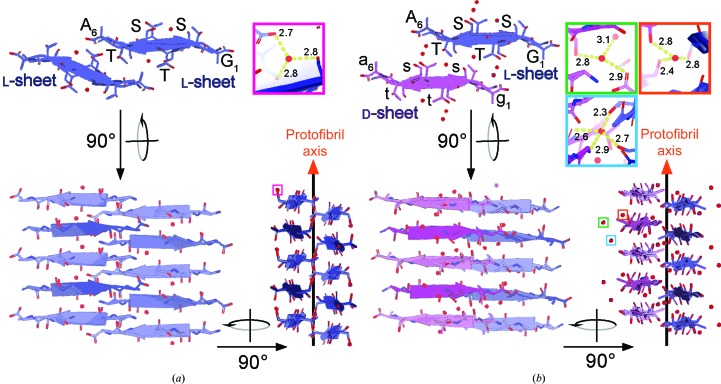
Views of protofibrils of l-GSTSTA (*a*) and racemic GSTSTA (*b*) represented by a pair of sheets with a view down the protofibril axis; both structures were derived by MicroED. A 90° rotation shows a side view of the protofibril with strands stacked along each sheet in an antiparallel fashion. Another 90° rotation shows a side view of the protofibril along the strand axis, showing a buckling of each sheet owing to the tilting of strands away from or towards the protofibril axis. Chains are colored such that blue represents l-peptides while magenta represents d-peptides. Lighter and darker shades of each color differentiate the orientations of strands within a sheet. Ordered waters found in each asymmetric unit are indicated by colored squares that correspond to the insets of matching colors. The insets show magnified views of each water molecule, with hydrogen bonds represented by yellow dashed lines and labeled with their corresponding distances in Å.

l-GSTSTA assembles into antiparallel in-register β-sheets that mate to form a protofibril (Fig. 4 ▸ *a* and Supplementary Figs. S12 and S13). The sheets are buckled, compressing the fibril along its length with strands spaced approximately 4.6 Å apart (Fig. 4 ▸ *a* and Supplementary Fig. S14), closer than the typical 4.7–4.8 Å spacings seen in amyloid protofibrils (Sawaya *et al.*, 2007 ▸). This spacing equates to half of the l-GSTSTA cell edge along the *a* axis: approximately 9.2 Å (Supplementary Table S1). To accommodate this compression, the strands tilt approximately 17° with respect to the fibril axis in alternating directions along a sheet, allowing the amides to lie askew from the fibril axis (Supplementary Fig. S14) while maintaining hydrogen bonding along the protofibril axis (Supplementary Table S3). Side chains between neighboring sheets tightly interdigitate to create a close packing within the fibril (Supplementary Fig. S12); the inter-sheet distances range from 5 to 7 Å. The interface created at the fibril core is small, with 229 Å^2^ of buried surface area, but shows a relatively high degree of shape complementarity (*S*_c_ = 0.75; Lawrence & Colman, 1993 ▸). The l-GSTSTA protofibril appears tightly restrained within the crystal structure, as shown by a mean *B* factor of 0.92 Å^2^. The modeled water molecule also appears to be well ordered, particularly in the structure of l-GSTSTA determined by MicroED, where it has a *B* factor of 3.28 Å^2^. The single coordinated water is hydrogen-bonded to Ser708, the C-terminus of a symmetry-related strand and the backbone of Thr709 in the mating sheet (Fig. 4 ▸, Supplementary Figs. S12 and S13, and Supplementary Table S4).

### 3.7. Structure of racemic GSTSTA   

In crystals of racemic GSTSTA, homochiral strands stack to form single-enantiomer antiparallel β-sheets (Fig. 4 ▸ *b* and Supplementary Fig. S13). Like the homochiral l-GSTSTA sheets, the racemic GSTSTA sheets are buckled, with adjacent strands spaced 4.6 Å apart along each sheet (Fig. 4 ▸ *b*). In the structure of racemic GSTSTA these sheets pack with alternating chirality, whereby each racemic GSTSTA protofibril is composed of one l-GSTSTA sheet and one d-GSTSTA sheet (Fig. 4 ▸ *b*). The packing of d-GSTSTA sheets against their l-GSTSTA mates in the racemic fibril differs from that seen in the homochiral fibrils of l-GSTSTA. An alignment of the two protofibrils shows d-GSTSTA sheets displaced by approximately 5.3 Å compared with their corresponding l counterparts in the homochiral fibril (Fig. 4 ▸ and Supplementary Fig. S15). As a result of this displacement, the sheets are spaced farther apart (7–8 Å) in the racemic GSTSTA protofibril (Fig. 4 ▸ and Supplementary Fig. S12).

The longer spacing between sheets in the racemic GSTSTA protofibril is associated with bridging waters at its core (Supplementary Fig. S12). These waters make extensive contacts along the protofibril, with each hydrogen-bonding to at least one residue (Fig. 4 ▸, Supplementary Fig. S13 and Supplementary Table S4). Notably, the racemic GSTSTA structure shows a distinct rotamer for Ser710, which appears bound to an ordered water, unlike its equivalent residue in the homochiral structure (Fig. 4 ▸, Supplementary S15 and Supplementary Table S4). One water (water 1; Supplementary Table S4) links Ser708 and Thr711 on the same d sheet while also coordinating Ser708 of the adjacent l sheet. This water is isolated from the other waters found within the structure. A small network of waters near the protofibril core links the carboxylate of one strand to Thr711 of a symmetry-related strand (Fig. 4 ▸, Supplementary Table S4). As in the structure of homochiral l-GSTSTA, the peptide atoms and bound waters in racemic GSTSTA show low *B* factors.

## 4. Discussion   

Ice nucleation by *P. syringae* is linked to the expression of surface proteins, including InaZ (Wolber *et al.*, 1986 ▸). While full-length InaZ and InaZ fragments help to nucleate ice (Green & Warren, 1985 ▸; Kobashigawa *et al.*, 2005 ▸), individual InaZ repeats do not (Han *et al.*, 2017 ▸). However, at the high concentrations required for crystallization, GSTSTA repeats self-assemble into a protofibrillar structure of corrugated β-sheets (Supplementary Fig. S14). Similar structures are formed by both racemic GSTSTA and l-GSTSTA, and both contain ordered waters bridging tightly packed antiparallel β-sheets (Fig. 4 ▸, Supplementary Figs. S12 and S13). These waters may play a role in helping to stabilize the GSTSTA protofibril or could act as bridges or templates for solvent ordering at low temperatures. While we have no evidence to suggest that GST*X*T(A/S) repeats facilitate the formation of amyloid-like InaZ protofibrils, our structures of GSTSTA present an opportunity to analyze the interactions between polar residues in InaZ repeats and ordered solvent molecules at atomic resolution.

The structures of entantiomerically pure and racemic GSTSTA present a platform for the comparison of homochiral and racemic amyloid protofibrils (Supplementary Fig. S16). To evaluate the packing of each GSTSTA protofibril, we look at the categorization of strand packing in amyloid fibrils through homosteric zipper classes, which were first proposed by Sawaya *et al.* (2007 ▸) and later by Stroud (2013 ▸). Many of these classes have been experimentally observed in amyloid crystals (Nelson *et al.*, 2005 ▸; Sawaya *et al.*, 2007 ▸). Homochiral GSTSTA forms a class 8 zipper in which two in-register, antiparallel β-sheets meet, related by a 180° rotation normal to the protofibril growth axis (Sawaya *et al.*, 2007 ▸; Stroud, 2013 ▸). The racemic GSTSTA structure resembles a class 8 zipper but is distinct in that two sheets of opposite handedness come together to form the protofibril (Supplementary Fig. S16). Because of this similarity to a class 8 zipper, we label this arrangement class 8 bar (Supplementary Fig. S16).

The increased propensity for crystallization by racemic mixtures could be exploited to facilitate the growth of amyloid crystals. The symmetry present in racemic amyloid crystals would have to accommodate the packing of homochiral protofibrils into the racemic structure or allow the formation of racemic protofibrils (Yeates & Kent, 2012 ▸), as is the case with GSTSTA. Our experiments in high-throughput crystallo­graphic trials of GSTSTA confirm the expected higher propensity for crystallization of racemic mixtures (Yeates & Kent, 2012 ▸; Supplementary Fig. S3), yielding a high number of conditions that contain submicrometre-sized crystals suitable for MicroED. The facile determination of *ab initio* structures from these crystals demonstrates how MicroED combined with solid-phase polypeptide synthesis (Dawson *et al.*, 1994 ▸; Merrifield, 1986 ▸) can expand the reach of racemic crystallo­graphy to submicrometre-sized crystals.

## Supplementary Material

PDB reference: racemic GSTSTA, X-ray structure, 6m7m

PDB reference: MicroED structure, 6m9j

PDB reference: l-GSTSTA, X-ray structure, 6eex

PDB reference: MicroED structure, 6m9i

Supplementary Tables and Figures.. DOI: 10.1107/S2052252518017621/fq5004sup1.pdf
