# Supplementary material for: Homochiral and racemic MicroED structures of a peptide repeat from the ice-nucleation protein InaZ
Source: IUCrJ. 2019 Jan 24;6(Pt 2):197–205. doi: 10.1107/S2052252518017621 (PMC6400192; doi:10.1107/S2052252518017621)
Supplement: Supplementary file 1 [file m-06-00197-sup1.pdf]

IUCrJ

Volume 6 (2019)

Supporting information for article:

Homochiral and racemic MicroED structures of a peptide repeat from the ice-nucleation protein InaZ

Chih-Te Zee, Calina Glynn, Marcus Gallagher-Jones, Jennifer Miao, Carlos G. Santiago, Duilio Cascio, Tamir Gonen, Michael R. Sawaya and Jose A. Rodriguez

Table S1 Crystallographic data collection and refinement statistics.

Values in parentheses are for the highest-resolution shell. All modelled waters have an occupancy of 1 after refinement.

Crystal	L-GSTSTA		Racemic GSTSTA	
Data collection				
Beam Type	x-ray	electron	x-ray	electron
Space group	$P2_12_12_1$	$P2_12_12_1$	$P2_1/C$	$P2_1/C$
Cell dimensions				
a, b, c (Å)	9.21 11.98 22.80	9.19 11.89 22.43	15.23 9.29 21.06	14.03 9.22 20.77
α, β, γ (°)	90.0 90.0 90.0	90.0 90.0 90.0	90.0 108.2 90.0	90.0 104.5 90.0
Resolution limit (Å)	1.1 (1.14–1.1)	0.90 (0.93–0.90)	1.1 (1.14–1.1)	0.9 (0.93–0.90)
Wavelength (Å)	0.979	0.0251	0.979	0.0251
No. of crystals merged	1	3	1	2
R_{merge}	0.125 (0.241)	0.1618 (0.3651)	0.076 (0.163)	0.128 (0.352)
R_{meas}	0.136 (0.265)	0.185 (0.422)	0.094 (0.206)	0.151 (0.411)
R_{pim}	0.053 (0.108)	0.084 (0.200)	0.053 (0.123)	0.078 (0.207)
I/σ_I	10.6 (6.7)	5.52 (2.60)	6.99 (2.98)	5.46 (2.47)
$CC_{1/2}$	0.99 (0.99)	0.99 (0.74)	0.99 (0.99)	0.99 (0.96)
Completeness (%)	91.7 (72.7)	86.4 (80.0)	93.7 (82.4)	77.4 (78.4)
No. reflections	7054 (516)	7494 (624)	5980 (477)	9884 (963)
No. unique reflections	1079 (80)	1750 (164)	2097 (191)	2906 (304)
Multiplicity	6.5 (6.5)	4.3 (3.8)	2.9 (2.5)	3.4 (3.2)
Refinement				
Resolution range (Å)	11.4-1.1 (1.14-1.1)	7.1-0.90 (0.94-0.9)	14.4-1.1 (1.14-1.1)	7.6-0.90 (0.94-0.9)
No. of Reflections (work)	968 (72)	1573 (16)	1873 (168)	2607 (274)
R-work	0.061 (0.078)	0.217 (0.305)	0.199 (0.342)	0.233 (0.311)
R-free	0.069 (0.087)	0.232 (0.287)	0.237 (0.311)	0.252 (0.421)
CC(work)	0.997 (0.995)	0.940 (0.786)	0.989 (0.897)	0.953 (0.862)
CC(free)	0.987 (1.000)	0.950 (0.744)	0.989 (0.725)	0.953 (0.706)
No. of hydrogen atoms	31	31	31	31
No. of non-hydrogen atoms	37	37	40	39
Peptide	36	36	36	36
Water	1	1	4	3
B-factors (Å^2)				
Peptide	1.77	0.92	9.17	2.40
Water	4.78	3.28	15.1	20.81

Rms deviations				
RMS(bonds, Å)	0.007	0.009	0.008	0.008
RMS(angles, °)	1.55	1.06	0.76	1.12

Table S2 Data reduction statistics for homochiral and racemic GSTSTA crystals for microfocal x-ray diffraction and MicroED.

Resolution	Number of Reflections				R-Factor					
Limit	Observed	Unique	Possible	Completeness	Observed	Expected	Compared	I/σ	Rmeas	CC(1/2)
**Racemic GSTSTA**										
**MicroED**										
1.79	1149	357	471	75.8%	8.4%	9.1%	1146	9.94	10.0%	99.1*
1.43	1230	363	470	77.2%	13.4%	12.3%	1226	7.67	15.8%	98.1*
1.25	1248	364	473	77.0%	15.0%	15.6%	1242	6.07	17.6%	98.9*
1.13	1238	358	459	78.0%	14.5%	15.4%	1233	5.41	17.1%	99.2*
1.05	1252	360	462	77.9%	16.9%	18.5%	1242	5.20	19.7%	98.1*
0.99	1271	360	466	77.3%	24.0%	26.3%	1262	3.99	27.9%	98.2*
0.94	1288	366	458	79.9%	35.4%	40.1%	1283	3.12	41.5%	90.3*
0.90	1208	378	487	77.6%	36.3%	44.3%	1149	2.45	42.6%	94.3*
**total**	**9884**	**2906**	**3746**	**77.6%**	**12.8%**	**13.6%**	**9783**	**5.46**	**15.1%**	**99.1***
**Microfocal x-ray**										
1.79	1484	506	514	98.4%	6.7%	7.3%	1442	12.25	8.2%	99.5*
1.43	1364	481	508	94.7%	9.0%	9.3%	1315	7.94	11.1%	99.3*
1.25	1355	469	502	93.4%	13.1%	13.3%	1309	5.15	15.8%	99.3*
1.13	1459	509	541	94.1%	15.2%	16.0%	1415	3.66	18.7%	99.5*
1.10	318	132	164	80.5%	19.8%	18.7%	292	2.70	25.2%	99.4*
**total**	**5980**	**2097**	**2229**	**94.1%**	**7.6%**	**8.2%**	**5773**	**6.99**	**9.4%**	**99.6***
**L-GSTSTA**										
**MicroED**										
1.80	902	237	298	79.5%	9.8%	12.2%	896	9.12	11.3%	99.2*
1.43	972	229	261	87.7%	16.4%	15.8%	965	7.25	18.6%	96.7*
1.25	974	223	254	87.8%	20.2%	19.3%	965	6.32	23.1%	94.4*
1.14	952	219	251	87.3%	21.0%	21.6%	949	5.46	23.8%	95.5*
1.05	992	218	245	89.0%	22.6%	23.7%	981	4.96	25.4%	96.1*
0.99	944	211	243	86.8%	22.8%	27.0%	938	4.25	25.8%	97.2*
0.94	972	216	241	89.6%	35.4%	39.1%	962	3.42	40.3%	70.2*
0.90	800	205	250	82.0%	37.8%	45.7%	775	2.61	43.5%	71.0*
**total**	**7508**	**1758**	**2043**	**86.0%**	**16.2%**	**17.9%**	**7431**	**5.50**	**18.5%**	**99.2***
**Microfocal x-ray**										
1.80	1768	290	304	95.4%	8.7%	9.7%	1757	14.25	9.7%	98.8*
1.43	1681	254	270	94.1%	14.5%	14.3%	1677	10.57	15.7%	98.8*
1.25	1666	246	263	93.5%	18.7%	17.7%	1662	9.15	20.3%	98.0*
1.134	1423	220	239	92.1%	21.1%	19.4%	1413	7.92	23.0%	97.2*
1.10	540	83	116	71.6%	23.7%	23.5%	539	6.86	26.1%	98.4*
**total**	**7078**	**1093**	**1192**	**91.7%**	**12.5%**	**12.7%**	**7048**	**10.41**	**13.6%**	**98.8***

Table S3 Hydrogen bonding between adjacent strands in homochiral L-GSTSTA sheets.

Peptide donor and acceptors are denoted as: chain name followed by a three-digit residue number and the associated three letter code. This table includes only unique hydrogen bonds between strands in a single beta sheet along the protofibril axis. All distances are measured in Ångströms, angles in degrees.

Donor	Atom	Acceptor	Atom	DA dist.	Type	CA to	DHA	H-A Dist.	H-A-AA	D-A-AA
B707-Gly	N	A712-Ala	O	2.80	MM	5.1	165.5	1.80	139.8	144.7
A712-Ala	N	B707-Gly	O	2.95	MM	5.1	153.1	2.03	140.6	142.1
B709-Thr	N	A710-Ser	O	2.97	MM	5.2	154.6	2.04	139.6	146.2
A710-Ser	N	B709-Thr	O	2.82	MM	5.2	151.3	1.90	147.2	157.0
A709-Thr	OG1	B710-Ser	OG	3.40	SS	4.1	153.7	2.48	94.0	99.2
B711-Thr	N	A708-Ser	O	2.89	MM	5.0	144.8	2.01	128.8	139.2
A708-Ser	N	B711-Thr	O	2.92	MM	5.0	154.7	1.98	137.6	138.0
B711-Thr	OG1	A708-Ser	OG	2.68	SS	5.0	165.0	1.70	130.4	125.4

Table S4 Hydrogen bonds of modelled waters in homochiral and racemic GSTSTA structures.

Peptide donor and acceptor names are abbreviated as the chain name followed by a three-digit residue number, a dash, and the associated three letter code. All hydrogen bonds in the table include a water as a donor or acceptor and are restricted to waters found in one asymmetric unit. All distances are measured in ångströms, angles in degrees. Only hydrogen bonds with distances below 3.2 Å are listed. The list includes potential hydrogen bonding partners, though not all might be satisfied at a time for a given atom.

Donor Name	Atom Type	Acceptor Name	Atom Type	DA dist.	D-A-AA angle
**L- GSTSTA, X-Ray**					
Water 1	O	A709-Thr	O	2.84	113.9
Water 1	O	C712-Ala	O	2.78	109.1
B708-Ser	OG1	Water 1	O	2.72	
**Racemic GSTSTA, X-Ray**					
Water 1	O	A709-Thr	O	2.93	121.1
B708-Ser	OG1	Water 1	O	2.82	
B710-Ser	OG1	Water 1	O	2.85	
Water 2	O	G712-D-Ala	O	2.89	107.2
Water 2	O	Water 3	O	2.71	
E711-D-Thr	OG1	Water 2	O	2.93	
Water 3	O	Water 4	O	2.94	
Water 3	O	E712-D-Ala	O	2.86	102.6
H707-Gly	N	Water 3	O	2.80	
C709-Thr	OG1	Water 3	O	2.67	
Water 4	O	C708-Ser	O	2.78	122.3
Water 4	O	B711-Thr	O	2.89	127.8
H707-Gly	N	Water 4	O	2.90	
C709-Thr	OG1	Water 4	O	3.02	
**L-GSTSTA, MicroED**					
Water 1	O	D712-Ala	O	2.66	112.7
Water 1	O	B709-Thr	O	2.76	113.3
A708-Ser	OG1	Water 1	O	2.76	
**Racemic GSTSTA, MicroED**					
Water 1	O	E711-D-Thr	OG1	2.81	126.9
D708-D-Ser	OG1	Water 1	O	2.42	
B708-Ser	OG1	Water 1	O	2.76	
Water 2	O	Water 3	O	3.05	
Water 2	O	G712-D-Ala	O	2.87	107.4
E711-D-Thr	OG1	Water 2	O	2.76	
Water 3	O	H712-D-Ala	O	2.62	108.1
H707-Gly	N	Water 3	O	2.93	
C709-Thr	OG1	Water 3	O	2.34	
B710-Ser	OG1	Water 3	O	2.71	

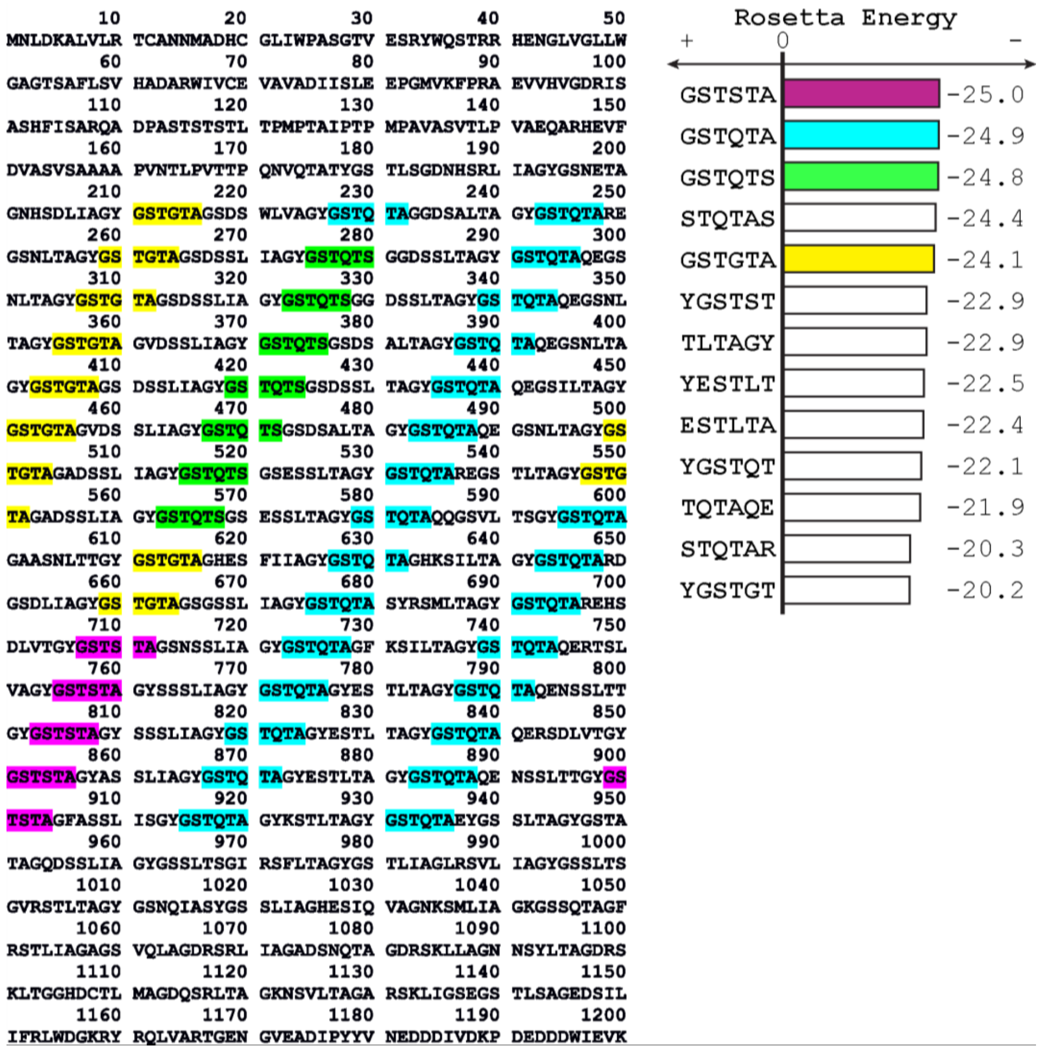

Figure S1 Sequence of the ice nucleation protein InaZ is shown with its degenerate hexameric repeats highlighted as follows: GSTGTA (yellow), GSTQTA (cyan), GSTSTA (magenta), and GSTQTS (green). The propensity for the hexamers to form steric zippers is shown on the left as Rosetta energy scores, determined by ZipperDB (Goldschmidt *et al.*, 2010). This list of repeats is limited to those with Rosetta energy lower than -20 that containing at least two threonine residues and appear with frequency greater than or equal to five across the InaZ sequence.

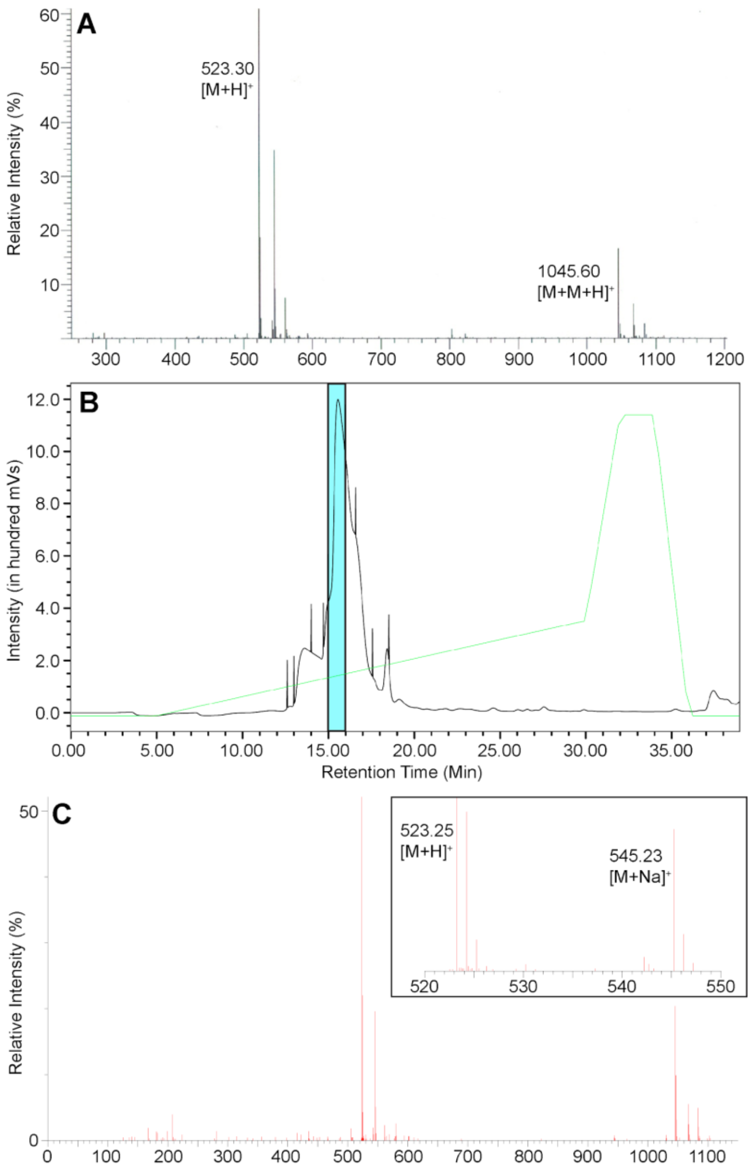

Figure S2 The mass trace of L-GSTSTA (A), purchased from Genscript, shows a $[M+H]^+$ peak of 523.30 g/mol (expected 523.22) and a dimer $[M+M+H]^+$ peak of 1045.6 g/mol (expected 1045.44). D-GSTSTA was synthesized and purified in-house by reverse-phase HPLC (B). The cyan shaded region highlights the HPLC fraction collected and lyophilized for crystallization experiments. The mass spectrum of D-GSTSTA (C) shows a $[M+H]^+$ peak of 523.24 g/mol (expected 523.22) and a $[M+Na]^+$ peak of 545.23 g/mol (expected 545.22).

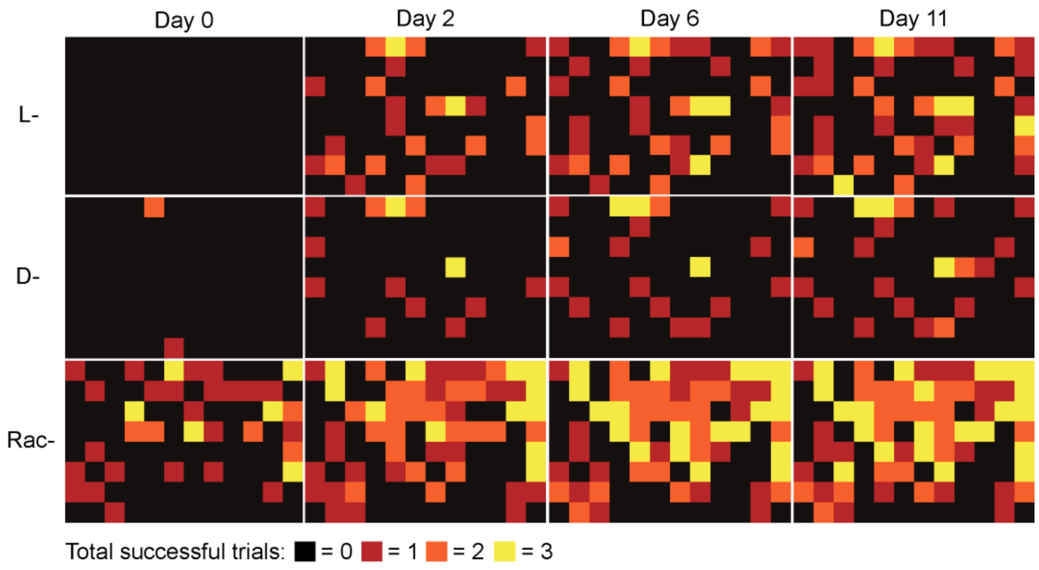

Figure S3 The number of successful crystallization trials in the 96-well crystal screens for L-GSTSTA, D-GSTSTA, and racemic GSTSTA were monitored and converted to heatmaps. Each well contained three hanging drops, each with different protein to buffer ratios. A count of crystals found in each of these three conditions was given a score from 0 to 3. No crystals in drops (black), one drop with crystals (red), two drops with crystals (orange), crystals in all three drops (yellow). The initial time point was three hours after setting up the screens (day 0), with data points collected up to 11 days post-setup.

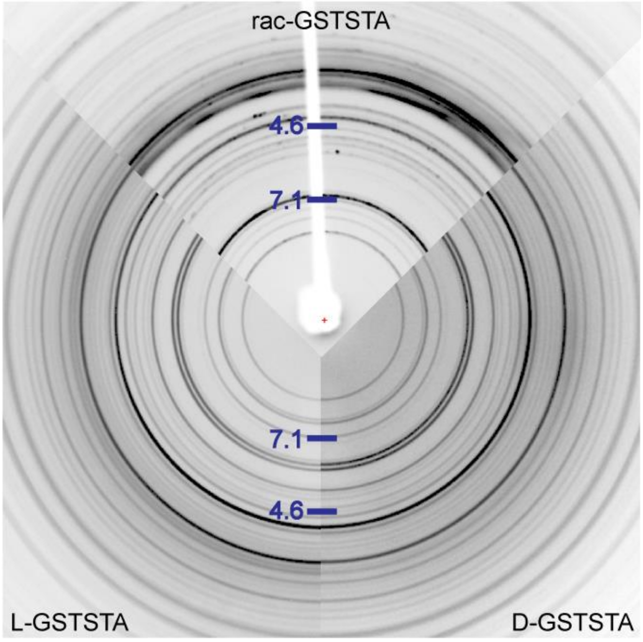

Figure S4 Comparison of powder diffraction patterns measured from L-GSTSTA (left), D-GSTSTA (right), and racemic GSTSTA (top) slurries show differences across all resolutions. Both patterns show faint rings at approximately 4.6Å, representing the approximate distance between strands along the fibril axis. The racemic pattern contains a prominent reflection at ~7.1Å, representative of overall sheet-to-sheet distances in its structure.

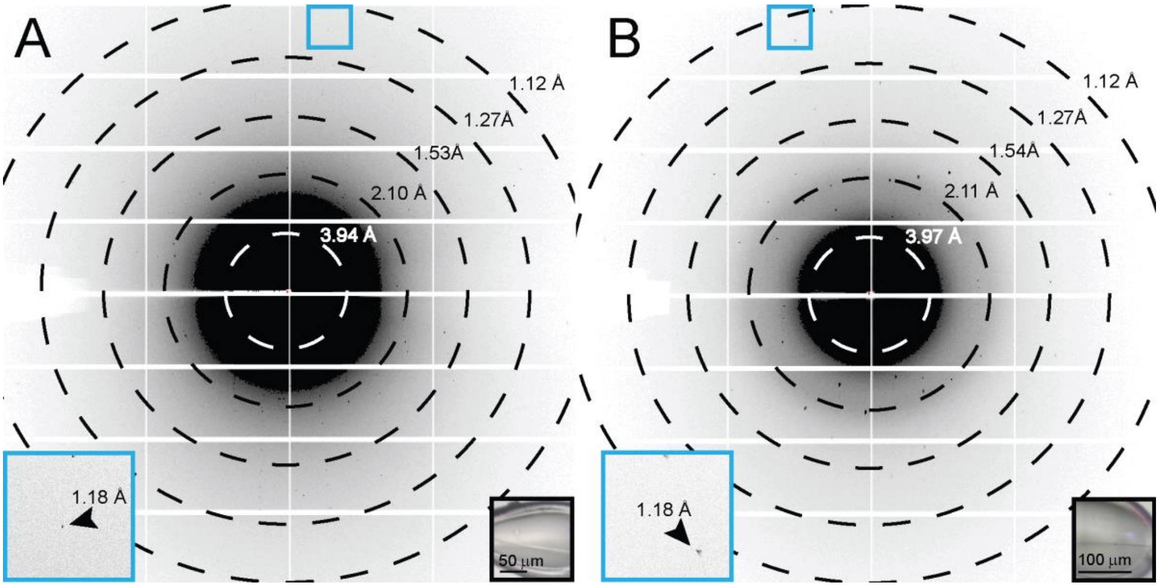

Figure S5 Single diffraction patterns of homochiral L-GSTSTA (A) or racemic GSTSTA (B) measured during continuous vector scanning, microfocus x-ray data collection. Each pattern corresponds to a 5° wedge (A) or 2.5° wedge (B) of reciprocal space. Black insets show in-line images of the crystals that were diffracted; blue squares correspond to magnified regions (blue insets) of the pattern that show diffraction near the detector edge at approximately 1.1Å resolution (black arrows). Resolution circles are indicated by rings; scale bars are 50 to 100 µm for L-GSTSTA and racemic GSTSTA, respectively.

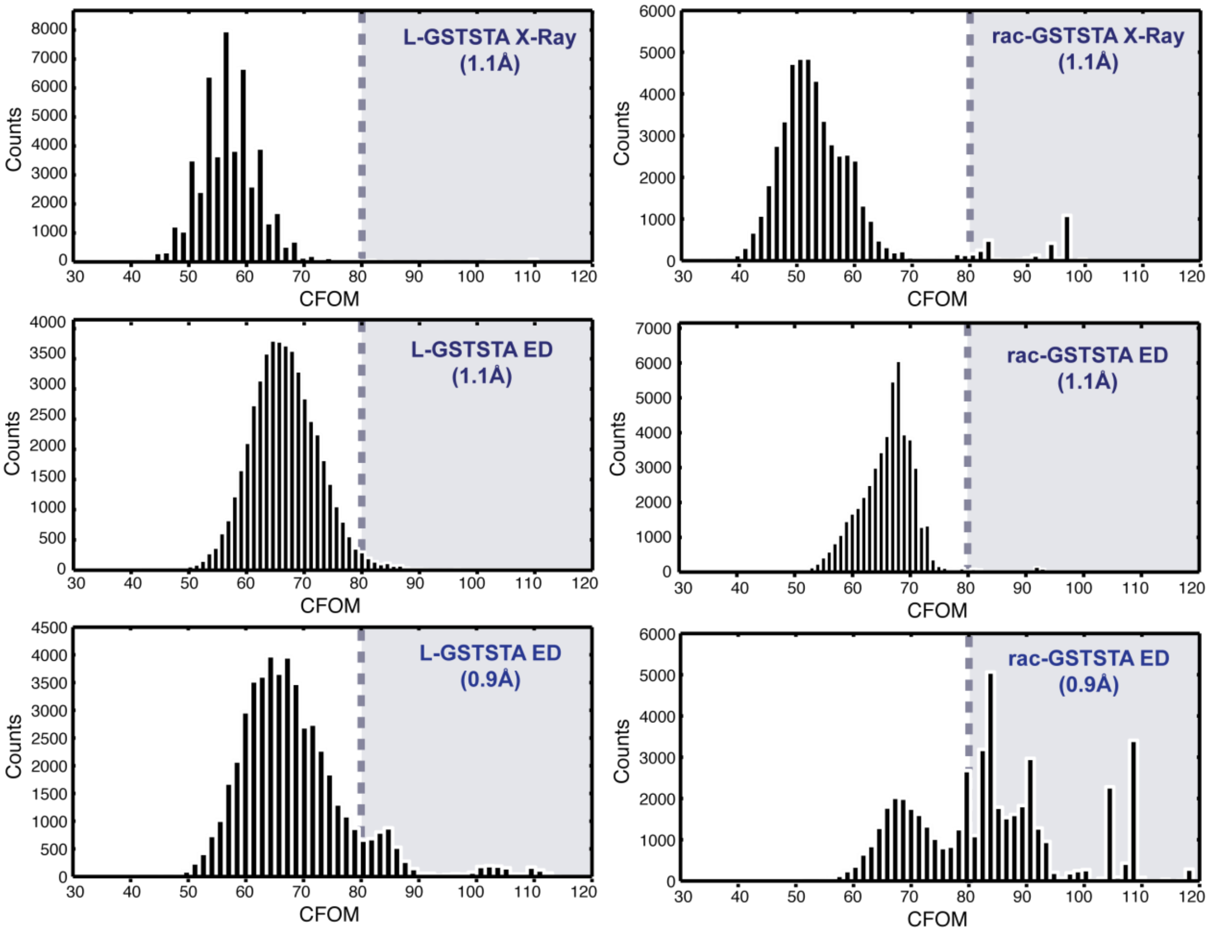

Figure S6 Histograms of the combined figure of merit (CFOM) scores for 50,000 trials by SHELXD indicate the approximate frequency of correct solutions. The shaded region in each plot, where CFOM scores are greater than 80, represents an area in which solutions have a high probability of being correct. Two sets of plots were generated from MicroED data: results of attempts using a truncated dataset that matches the resolution of the x-ray data (1.1Å) are shown in middle panels, while bottom panels show results of attempts using the measured resolution (0.9Å) for that data.

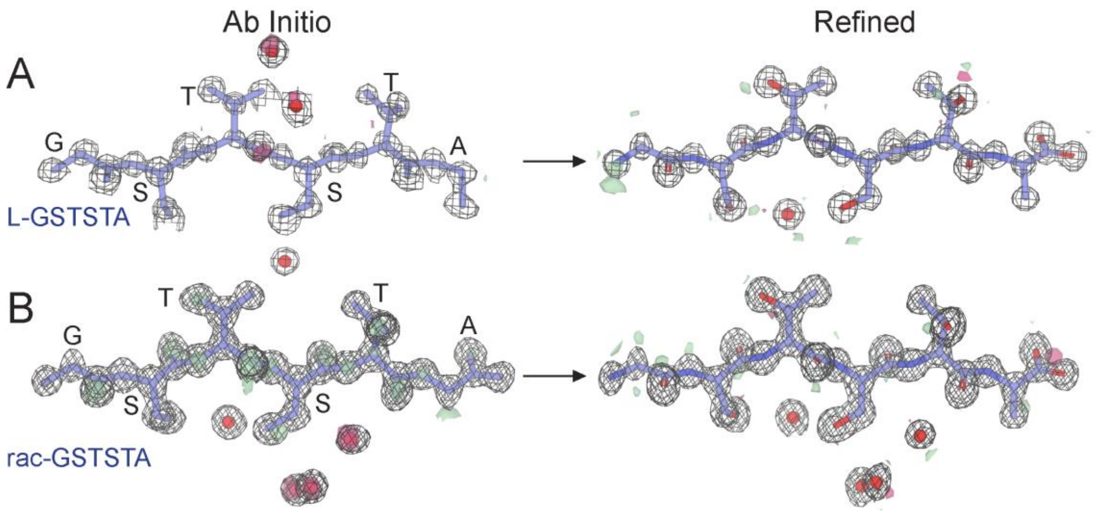

Figure S7 *Ab Initio* structures and electron density maps of L-GSTSTA (A) or racemic GSTSTA (B). Each map is in A is overlaid onto the initial atomic coordinates calculated by SHELXD from x-ray diffraction data. Each map in B is overlaid onto its corresponding refined model. The $2F_o$-F_c map represented by the black mesh is contoured at 1.2 σ. Green and red surfaces represent the F_o-F_c maps contoured at 3.0 and -3.0 σ. Modelled waters are present as red spheres.

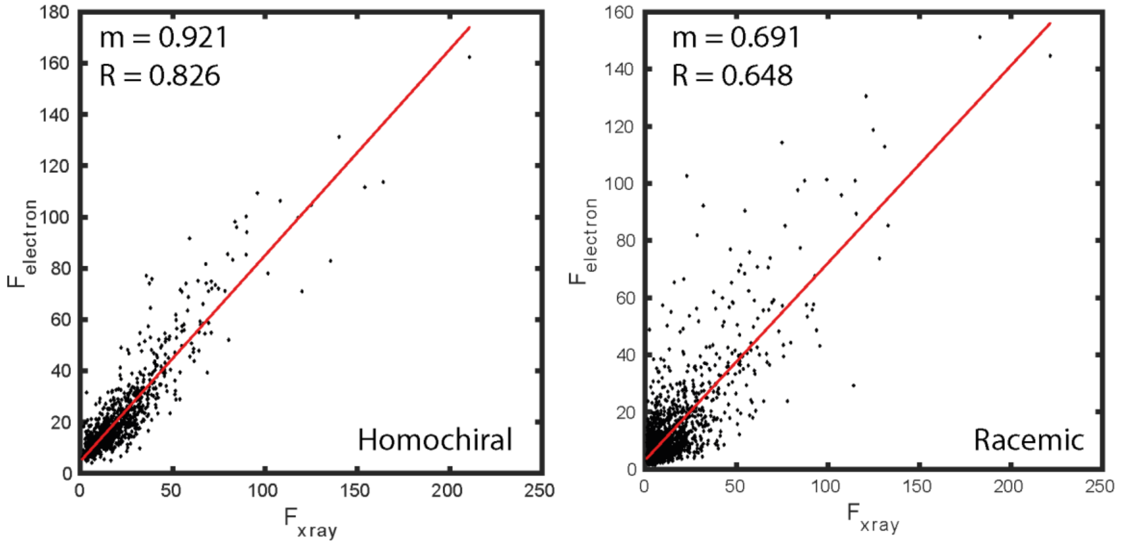

Figure S8 A plot of magnitudes (F) compared between reflections in datasets collected from homochiral crystals (left) or racemic crystals (right) by either electron or x-ray scattering shows a distribution that can be fit by linear regression, indicated by red lines with slope (m) and R-value.

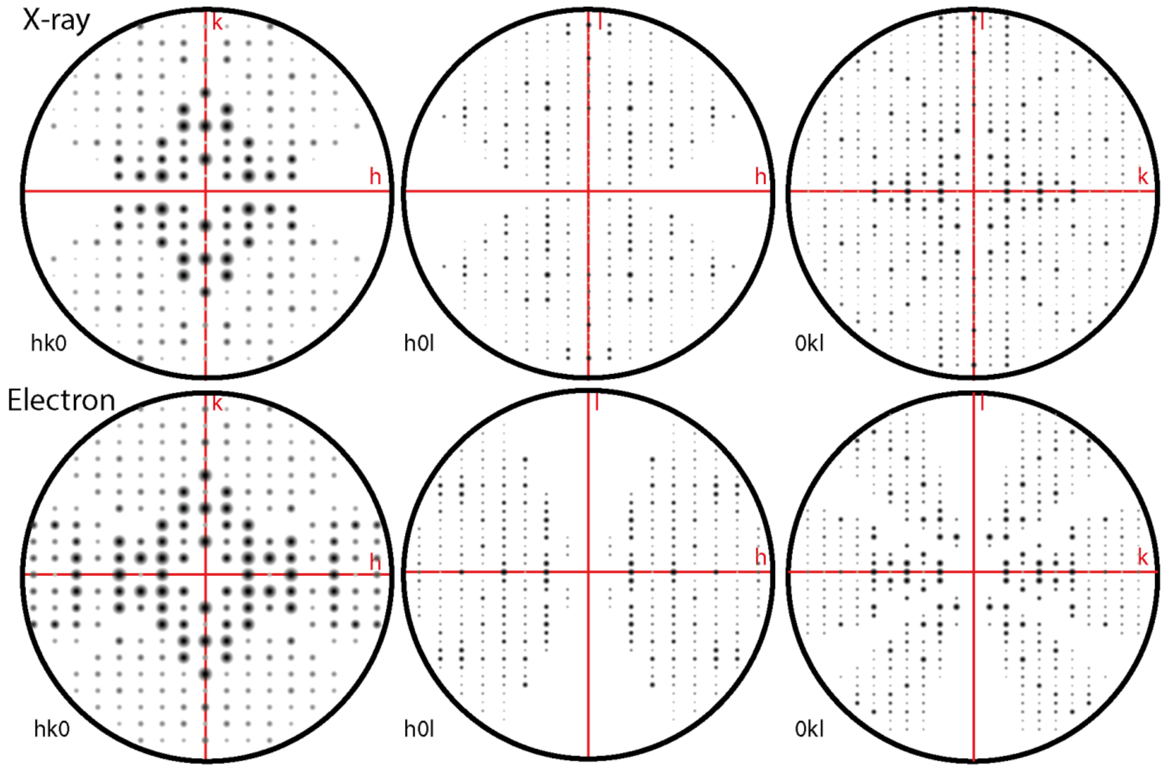

Figure S9 HKL Zone analysis for crystals of L-GSTSTA. Fourier magnitudes are shown as displayed by the HKL view software for reflections along principal zones of the reciprocal lattice. Zones where l=0 (left), k=0 (middle), h=0 (right) are shown for merged data collected by x-ray (top) and electron (bottom) diffraction. The black circle in each zone plot represents a resolution of 1.1Å; zone axes are labeled in red.

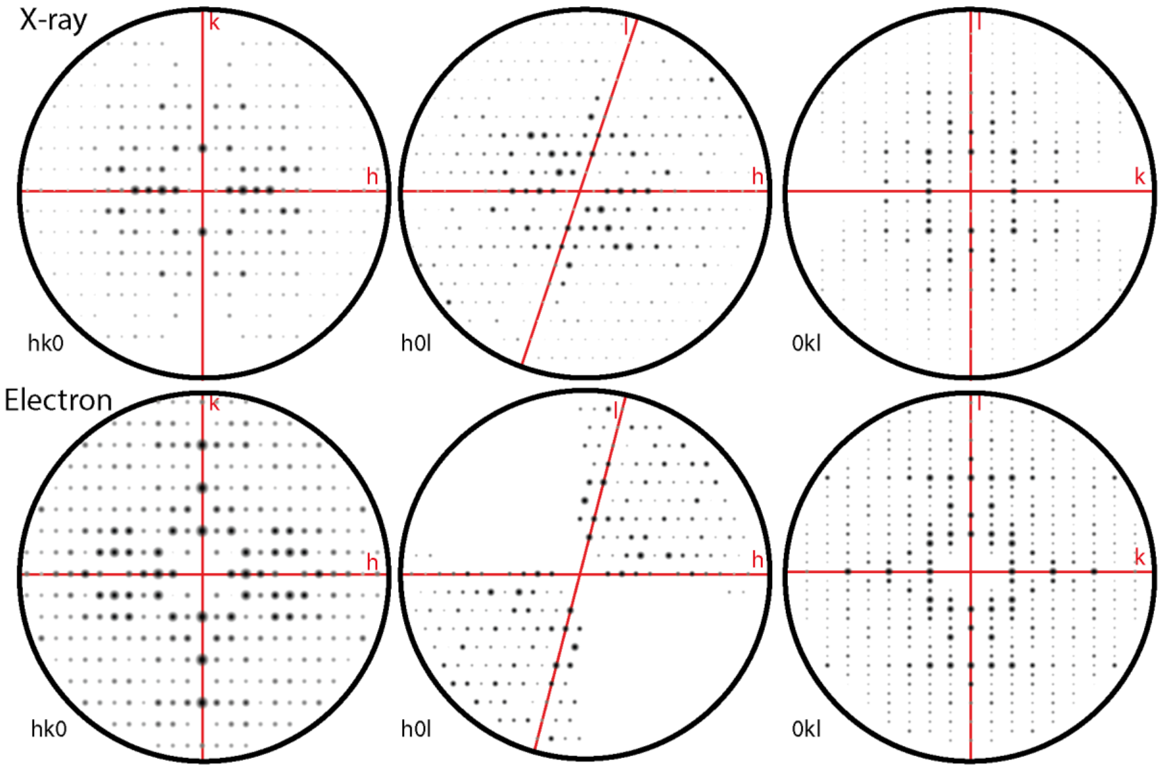

Figure S10 HKL Zone analysis for crystals of racemic GSTSTA. Fourier magnitudes are shown as displayed by the HKL view software for reflections along principal zones of the reciprocal lattice. Zones where l=0 (left), k=0 (middle), h=0 (right) are shown for merged data collected by x-ray (top) and electron (bottom) diffraction. The black circle in each zone plot represents a resolution of 1.1Å; zone axes are labeled in red.

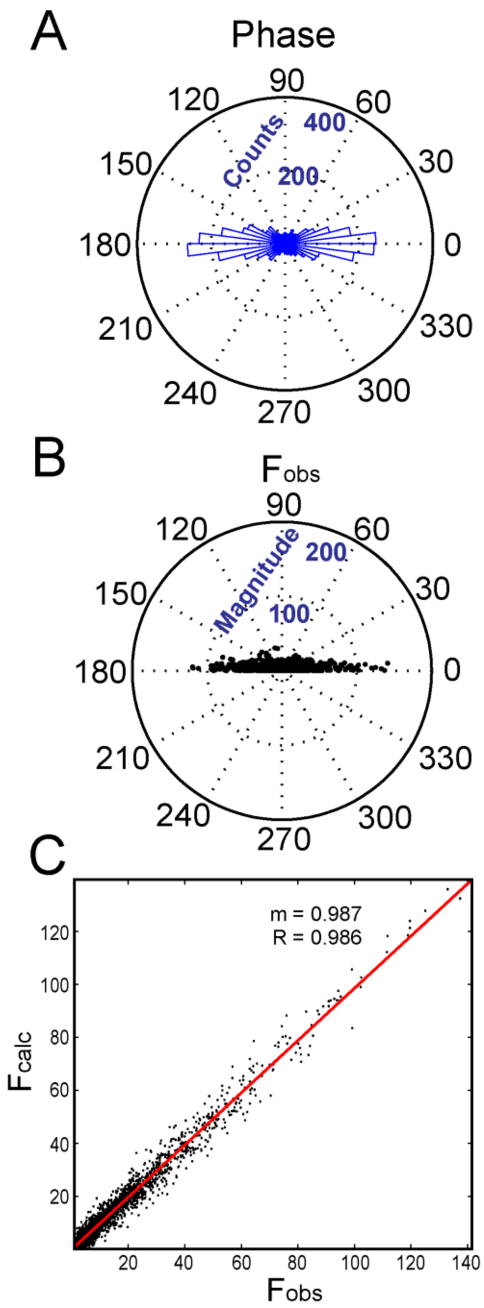

Figure S11 (A) The calculated phase associated with each reflection in the P1 refinement of racemic GSTSTA data obtained by x-ray diffraction was analyzed and plotted as a histogram along the unit circle. (B) The magnitude of each reflection is plotted as a function of the absolute value of its associated phase. (C) A plot of F_o vs. F_c values for each reflection in this data set shows a distribution that can be fit by linear regression, shown as a red line with slope m=0.987 and R-value 0.986.

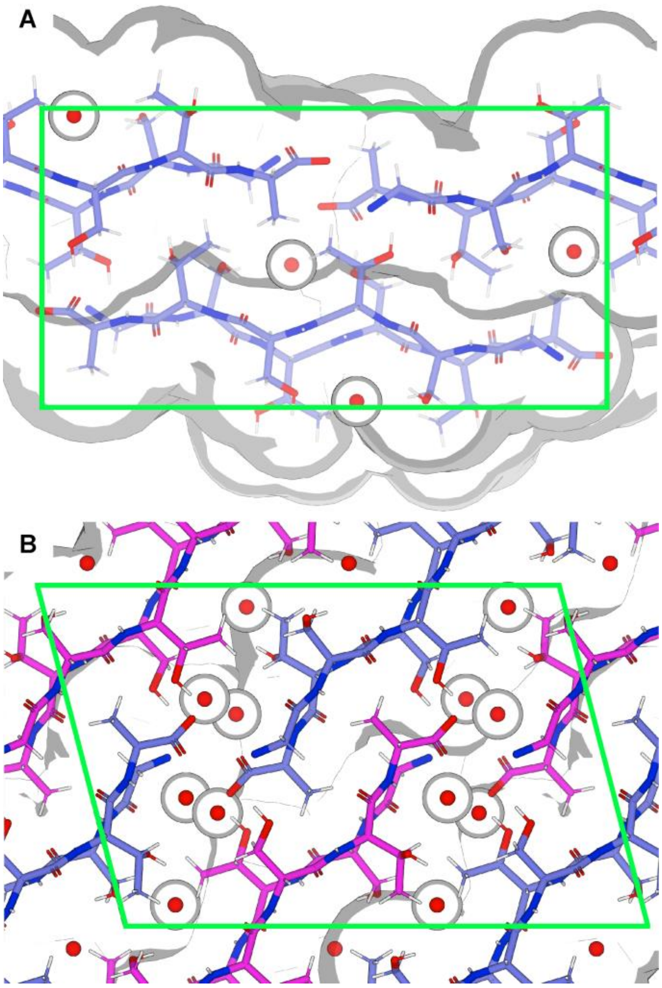

Figure S12 Views down the a-axis of L-GSTSTA (A) and racemic GSTSTA (B) structures are enclosed in green by their respective unit cells. L-GSTSTA strands are colored blue while D-GSTSTA strands are colored magenta. The volumes occupied by each structure are shown in white with edges defined by the shaded grey regions. Space-fill models represent the solvent accessible surface; ordered waters are represented by van der Waals radii of 1.4Å.

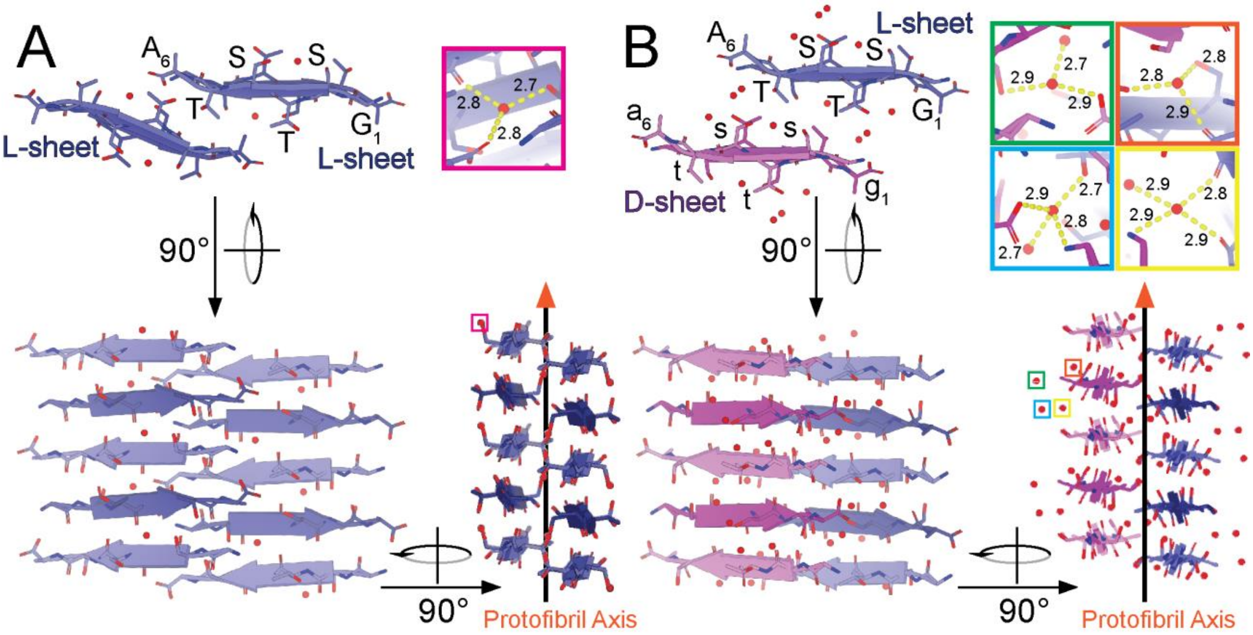

Figure S13 Views of protofibrils of L-GSTSTA (A) and racemic GSTSTA (B) represented by pair of sheets with a view down the protofibril axis; both structures derived by x-ray diffraction. A 90º rotation shows a side view of the protofibril with strands stacked along each sheet in an antiparallel fashion. Another 90º rotation shows a side view of the protofibril along the strand axis, showing a buckling of each sheet due to the tilting of strands away from or toward the protofibril axis. Chains colored such that blue represent L-peptides while magenta represents D-peptides. Lighter and darker shades of each color differentiate the orientation of strands within a sheet. Ordered waters found in each asymmetric unit are indicated by colored squares that correspond to insets of matching colors. Insets show magnified views of each water molecule with hydrogen bonds represented by the yellow dashed lines, labelled with their corresponding distances in Å

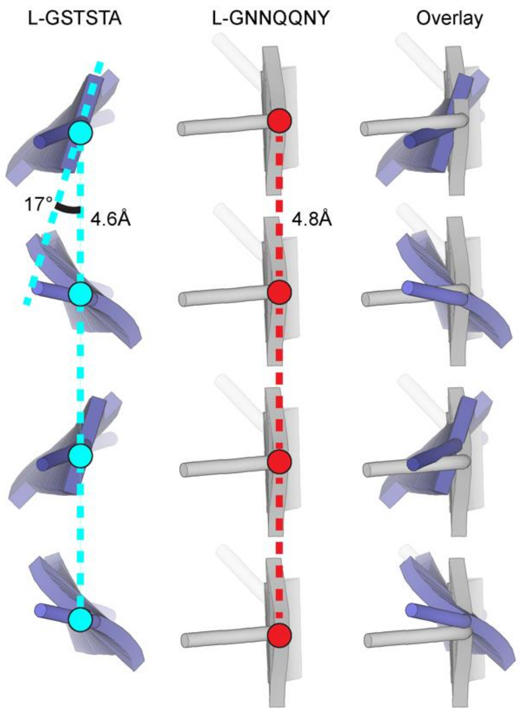

Figure S14 Beta sheets in the structure of L-GSTSTA have an inter-strand distance of 4.6Å with amide carbonyls angled away from the protofibril axis by approximately 17° (left). Canonical beta sheets formed by the yeast prion segment L-GNNQNNY (RCSB PDB: 1YJP) show inter-strand distance of 4.8Å and a near 0° deviation of amide hydrogen bonding down the protofibril axis (center) (Nelson *et al.*, 2005). An overlay (right) illustrates compaction of the L-GSTSTA sheet along its length compared to a sheet formed by L-GNNQQNY.

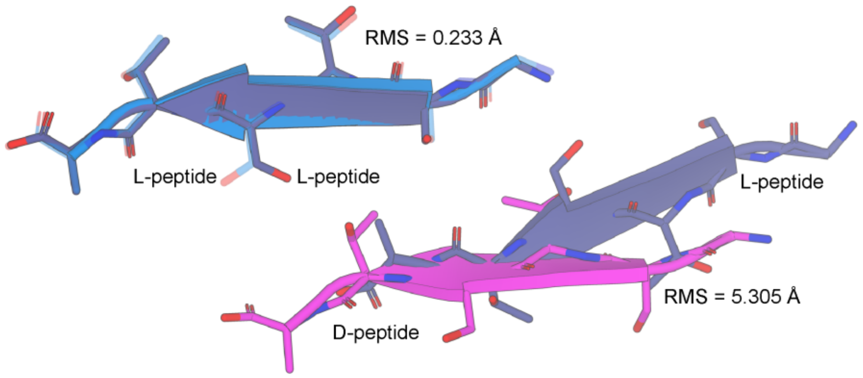

Figure S15 Pairs of mated strands representing the homochiral and racemic protofibrils of GSTSTA. Alignment of these protofibrils based on a common L-GSTSTA sheet shows a displacement of their paired sheet. The RMSD between the common L-GSTSTA sheets is 0.23 Å, while that between the pairing L and D sheets of the homochiral and racemic protofibrils is 5.3 Å. L-GSTSTA strands are colored blue and purple while D-GSTSTA is colored magenta.

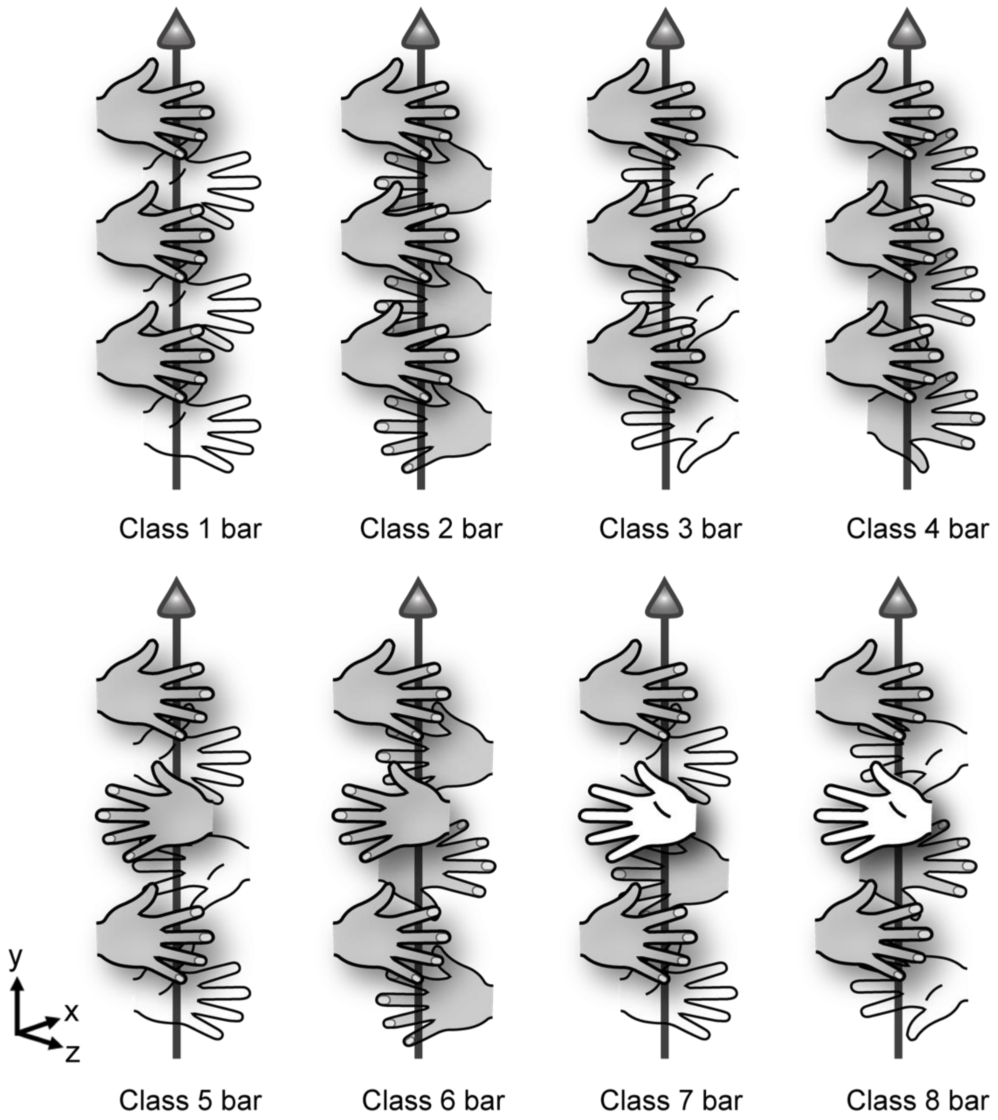

Figure S16 Eight new potential steric zipper classes are enabled by racemic assemblies. All new classes are based on those originally described in Sawaya *et al*. (Sawaya *et al.*, 2007) but now contain a mirror plane, a glide plane, or an inversion center. Strands are represented by left and right hands, each equivalent to the enantiomers present in a zipper class. The asymmetry of side chains on either side of a strand is portrayed by the palm and back of each hand. The up or down orientation of the thumbs and the direction in which the fingers point indicates the direction of each strand. An arrow indicates the axis of fibril growth, which here is coincident with the y direction. While additional symmetry classes with racemic mixtures are possible, only eight are illustrated here.
